# Gene Delivery into Plant Cells for Recombinant Protein Production

**DOI:** 10.1155/2015/932161

**Published:** 2015-05-17

**Authors:** Qiang Chen, Huafang Lai

**Affiliations:** ^1^Center for Infectious Disease and Vaccinology, The Biodesign Institute, Arizona State University, 1001 S. McAllister Avenue, Tempe, AZ 85287, USA; ^2^School of Life Sciences, Arizona State University, Tempe, AZ 85287, USA

## Abstract

Recombinant proteins are primarily produced from cultures of mammalian, insect, and bacteria cells. In recent years, the development of deconstructed virus-based vectors has allowed plants to become a viable platform for recombinant protein production, with advantages in versatility, speed, cost, scalability, and safety over the current production paradigms. In this paper, we review the recent progress in the methodology of agroinfiltration, a solution to overcome the challenge of transgene delivery into plant cells for large-scale manufacturing of recombinant proteins. General gene delivery methodologies in plants are first summarized, followed by extensive discussion on the application and scalability of each agroinfiltration method. New development of a spray-based agroinfiltration and its application on field-grown plants is highlighted. The discussion of agroinfiltration vectors focuses on their applications for producing complex and heteromultimeric proteins and is updated with the development of bridge vectors. Progress on agroinfiltration in *Nicotiana* and non-*Nicotiana* plant hosts is subsequently showcased in context of their applications for producing high-value human biologics and low-cost and high-volume industrial enzymes. These new advancements in agroinfiltration greatly enhance the robustness and scalability of transgene delivery in plants, facilitating the adoption of plant transient expression systems for manufacturing recombinant proteins with a broad range of applications.

## 1. Introduction

The approval of the first plant-derived therapeutic enzyme for Gaucher's disease has demonstrated the promise of plant-based systems for recombinant protein (RP) production [1]. In addition to the traditional advantages in cost, scalability, and safety over current bioreactor-based production platforms, progress in glycoengineering and expression vector discovery has also allowed plants to produce RPs with specific glycoforms to enhance functionality and at unprecedented speed to control potential pandemics and fight bioterrorism [2].

The traditional strategy of producing RPs in plants is to create stable, transgenic lines of plants. The target transgene is integrated into the plant genome and the RP can be produced in successive generations [3]. To eliminate the long time frame of generating transgenic plants, transient expression systems have been developed. In this strategy, the transgene is not integrated into the plant genome but rather quickly directs the production of the RP while residing transiently within the plant cell. In addition to significantly shortening the production timeline, this strategy also enhances RP accumulation level by eliminating the “position effect” of variable expression caused by the random integration of transgene within the genome [4]. Besides its speed and high yield, the transient expression system also offers the versatility for producing personalized RPs, such as therapeutics for patient-specific cancers and vaccines against viruses that have rapid antigenic drift and/or multiple strains with unpredictable outbreaks. This flexibility also provides the “surge” capability to rapidly produce recombinant counteragents in a bioterrorism event. Since no transgenic plant is created, transient expression also addresses regulatory issues and public concerns for genetically modified organisms (GMOs). These advantages demonstrate the vast potential of transient expression as a preferred method for RP production in plants. However, scale-up of RP production by transient expression poses a bigger challenge than transgenic plants, because no genetically stable seed bank is produced and scale-up is no longer just a matter of increasing acreage to boost yield. To overcome this challenge, a scalable transgene delivery method must be developed for plant transient expression.

## 2. Methods of Transgene Delivery

The method of choice for introducing transgenes into plants depends on the expression vector and the host plants. These methods include direct delivery by gene gun and indirect delivery through using* Agrobacterium tumefaciens* or plant viruses [5].

### 2.1. Direct Delivery Methods

DNA or RNA can be directly introduced into plant cells via a so-called microprojectile bombardment method, also known as a gene gun or biolistics. In this method, the transgene is coated onto microgold or tungsten particles and fired into plant cells ballistically [6]. The advantage of this method resides in its versatility and a broad range of susceptible plants. It can be used to deliver transgene to both nuclear and chloroplast genomes. At least in theory, effective transgene delivery by biolistics is vector independent and can be applied to any plant host species [5].

### 2.2. Indirect Gene Delivery Methods

Indirect transgene delivery exploits the ability of plant virus or certain pathogenic agrobacteria species (e.g.,* A. tumefaciens*) that naturally transfer their genome (plant virus) or part of their tumor inducing plasmid (Ti plasmid) DNA (T-DNA) into plant cells (*Agrobacterium*). A transgene can enter plant cells as a by-product of viral infection if cloned into the full viral genome. Infection can be facilitated by rubbing plant tissue with a transgene carrying infectious viral particles or viral nucleic acids [3]. However, this method is only applicable to viruses or plant hosts that are susceptible to mechanical inoculation but not to those that require specialized insects for viral transmission.

Ti plasmids of* Agrobacterium* can be modified into delivery vectors by replacing pathogenic genes in T-DNA with transgenes; transgene transfer from agrobacteria to plant cells is accomplished through the natural interaction between* A. tumefaciens* and its plant hosts [5]. In contrast to biolistics, gene delivery by* A. tumefaciens* requires the cloning of transgene into a modified Ti plasmid and is restricted to dicotyledonous and a limited number of monocotyledonous plants [7]. However, delivery by* Agrobacterium* generally offers better efficiency, transgene expression, and inheritance than biolistics [5]. It is speculated that* Agrobacterium*-based delivery is advantageous because transgene copy numbers and integration into the genome are better controlled. The coevolution of* Agrobacterium* and its plant hosts may favor the integration of transgenes into genomic loci that are transcriptionally active, which leads to its high level of expression [7]. In transient expression, biolistics often cause severe tissue damage and effectively reduce the available biomass for RP production, making indirect delivery by* Agrobacterium* a preferred method. As a result, an* Agrobacterium*-based gene delivery via agroinfiltration has become the favorable gene delivery method for transient expression in plants [7, 8].

## 3. Agroinfiltration for Expression of Recombinant Proteins

On a per cell basis, the yield of a RP is usually higher in transient expression than that in transgenic plants [4]. The elimination of the “position effect” is responsible for this improvement as the transgene is no longer randomly inserted into genomic areas with variable transcriptional activity [9]. However, earlier gene delivery methods consisted of soaking leaf pieces in* Agrobacterium* culture in which only the cell layer on the edges may receive the transgene. This limits the efficiency and scalability of transient systems.

Agroinfiltration was invented to overcome this challenge. Because up to one-third of the leaf volume is intercellular space, it is possible to replace the air in these cavities with a suspension of* Agrobacterium* [1]. Thus, transgene-carrying agrobacteria are actively delivered into the intercellular space of the leaf tissue by agroinfiltration, allowing for the effective access of agrobacteria to most leaf cells and making the transfer of T-DNA a highly efficient event [8, 10].

### 3.1. Application and Scalability of Agroinfiltration

**Figure 1:**
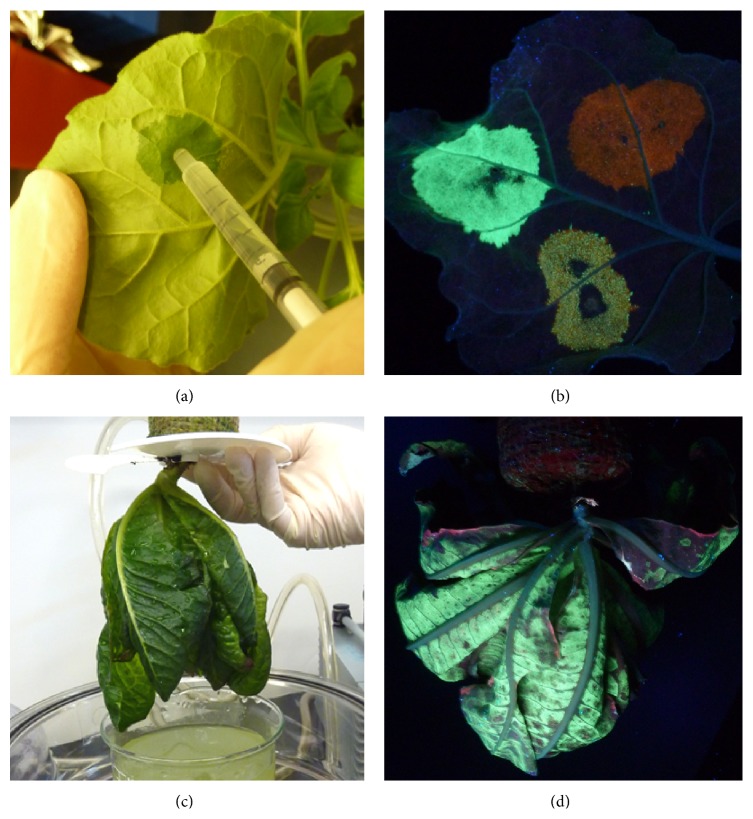
Transgene delivery by agroinfiltration into* N. benthamiana* and lettuce plants. Agrobacteria carrying the expression cassette of GFP or DsRed in geminiviral vectors were syringe-infiltrated into a* N. benthamiana* leaf (a) and GFP or DsRed expression was observed 4 days after infiltration under UV light (b). Similarly,* A. tumefaciens* cells harboring the expression cassette of GFP in a geminiviral vector were vacuum infiltrated into a lettuce plant (c) and GFP expression was examined 4 days after infiltration (d). The yellow spot in (b) indicates the leaf area that was coinfiltrated with agrobacteria carrying the expression cassette of GFP and DsRed.

The simplest method of agroinfiltration is syringe infiltration. In this method, transgene-carrying* Agrobacterium* in the infiltration medium is injected into the leaf with a needleless syringe (Figure 1(a)). Syringe infiltration offers the flexibility of introducing multiple transgene constructs into different areas, allowing multiple assays to be performed on a single leaf [8]. Thus, it has been used for studying plant-pathogen interactions, abiotic stresses, gene functional analysis, protein localization, and protein-protein interactions [8]. Syringe infiltration also has numerous applications for RP production. For example, it can be used to quickly examine the expression level of a RP under established conditions (Figure 1(b)). If further optimization of expression is necessary, its flexibility permits a quick assessment of various factors' effects on the yield, expression kinetics, and toxicity of the target protein. These factors include concentrations of* Agrobacterium* culture, different expression vectors, organelles favorable for RP accumulation, and the requirement for silencing suppressors. Once optimized, syringe infiltration can also be used to infiltrate several entire plants to rapidly obtain sufficient amounts (milligram level) of RPs for biochemical characterization and preclinical functional studies, as well as for developing purification schemes. Despite these utilities, only a few plant species are naturally amenable to syringe infiltration and the prospect of its scalability is highly limited [11].

A scalable agroinfiltration technology will enhance the competitiveness of transient expression systems with the traditional cell culture-based platforms for RP production. An agroinfiltration method using a vacuum chamber was developed for such purposes [5]. A prototype apparatus routinely used in the laboratory is best illustrative of the vacuum chamber method. First, aerial parts of plants are submerged into an* Agrobacterium* suspension. The submerged plants are then transferred into a desiccator that serves as the vacuum chamber. A pump provides the vacuum that exposes the submerged plants to a negative atmospheric pressure and draws the air out of the interstitial space of the leaves. Agroinfiltration is achieved when the vacuum is released, allowing agrobacteria in the medium to enter the intercellular space once occupied by the air (Figure 1(c)).

Vacuum infiltration can efficiently infiltrate plant species that are unamenable to syringe infiltration, effectively expanding the host range of agroinfiltration [12–14]. Furthermore, studies demonstrated that vacuum infiltration resulted in similar yield and temporal expression patterns for several RPs compared to that of syringe infiltration. This suggests that the results from the two agroinfiltration methods are mutually transferable; the simple syringe infiltration can accurately predict the expression pattern of a RP in scale-up settings. Not surprisingly, research has shown the superiority of vacuum infiltration in speed and robustness over syringe infiltration. For example, as the entire plant is subjected to agroinfiltration with vacuum, the expression of the RP can be detected in all leaves of the entire plant (Figure 1(d)). Even at bench scale, the time required for infiltrating a single 6-week-old* N. benthamiana* plant is significantly shortened 30 times by vacuum infiltration [15].

**Figure 2:**
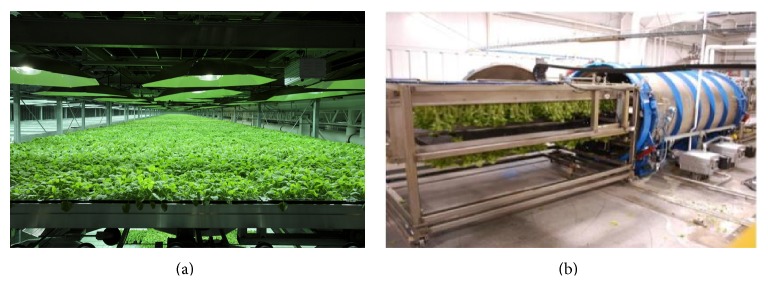
*N. benthamiana* plant growth (a) and agroinfiltration (b) at commercial production scale at Kentucky Bioprocessing LLC.

The scalability of vacuum infiltration has been examined for the production of RPs with biomedical applications. To test its scalability beyond the desiccator prototype, we designed a vacuum chamber that is able to accommodate 16 trays of plants per infiltration cycle. Results indicated that vacuum infiltration is highly scalable. Specifically, the accumulation level and the temporal expression patterns of virus-like particles (VLPs) of norovirus and monoclonal antibodies (MAbs) against West Nile virus (WNV) were not affected, regardless of whether they were produced under scale-up conditions or with the desiccator [16–19]. This pilot-scale system has enabled us to produce the norovirus vaccine candidate under current good manufacturing practice (cGMP) regulations which is sufficient both in quality and quantity for a phase I human clinical trial [16, 20, 21]. Biotechnology companies have further explored the scalability of this method. For example, a fully automated vacuum system was developed with the capability to agroinfiltrate up to 1.2 tons of plant biomass per day, allowing for the production of up to 75 g of MAb-based therapeutics per greenhouse lot (Figure 2) [1, 15, 22]. This process can be further scaled up, but its requirement of inverting plants grown in pots or trays may impose limitations on the ways the plants can be cultivated and may confine the use of vacuum infiltration to high-value RPs, such as vaccines and therapeutics.

For even larger scale RP production, especially for low-cost but high-volume RPs, it is desirable to develop new agroinfiltration technologies that allow gene delivery to whole plants without using a vacuum. Fortunately, as only nontransgenic plant material is used in transient expression, biomass can be generated in open fields by conventional agricultural practices without concerns for GMO. This allows the exploration of a spray-based agroinfiltration method to deliver transgene into field-grown plants. Initially, approximately 2% of leaf cells can receive and express the transgene through spray agroinfiltration [1]. New developments in this methodology include the use of surfactant and/or abrasives in the* Agrobacterium* suspension to enhance transfection and new* Agrobacterium* strains with super transfectivity [1, 23]. When improved spray agroinfiltration is combined with an expression vector that can generate a replicon with cell-to-cell movement capability, up to 90% of leaf cells can receive the transgene and express the target protein at high levels of 50% total soluble protein (TSP) [1]. This provides a simple and indefinitely scalable process of transgene delivery into field-grown plants, allowing transient expression on a large agricultural scale. The demonstration of large-scale agroinfiltration under the US Food and Drug Administration's (FDA) cGMP guidelines supports the regulatory compliance of this technology and extends its application to manufacture RPs with human biomedical interests. Collectively, these studies demonstrate that the value of vacuum and spray agroinfiltration lies in their enormous scalability potential, facilitating the adoption of plant transient expression-based systems for commercial manufacturing of RPs.

### 3.2. Vectors for Agroinfiltration

Agroinfiltration is versatile and can be performed with any vectors as long as they can replicate in* A. tumefaciens* and initiate T-DNA transfer with the help of virulent genes on chromosomes and/or another plasmid [8]. The earliest vectors used for agroinfiltration were transcriptional vectors such as pBIN19 or pCAMBIA, driven by nopaline synthase (pnos) or cauliflower mosaic virus (CaMV) 35S promoters (CaMV35S). While these transcriptional vectors are not as robust as later developed plant virus-based vectors, they do have a broad host range and can be used in almost all plant species. It is these vectors that demonstrated the superiority of transient expression in regard to the speed and RP yield over the traditional protein expression in transgenic plants [20, 24, 25].

The robust replication and/or transcription of plant viruses has led to the development of viral vectors for enhancing the RP yield [25]. Each type of plant virus offers its unique advantages and limitations as an agroinfiltration vector and may be useful for the production of a specific type of RP. For example, double-stranded DNA plant viruses such as CaMV are useful only for producing small RPs, because they have limited packaging capacity and can lose their genome functions when a fraction of their genomes are removed or substituted [25]. In contrast, single-stranded RNA viruses (e.g., tomato busy stunt virus (TBSV), tobacco mosaic virus (TMV), and potato virus X (PVX)) offer vectors for expressing large RPs, because they are more tolerant to large gene substitutions and insertions [25]. However, these RNA-based vectors have to be generated by an unscalable* in vitro* process.

“Deconstructed” viral vectors represent a new generation of vectors that combine the robustness of full viral vectors and the versatility of nonviral vectors. The elimination of unnecessary or unbeneficial genomic components during viral deconstruction significantly reduces the size of the replicon, allowing the insertion of larger transgenes while maintaining the robustness of viral replication and transcription. Deconstructed RNA viral vectors can be delivered in the form of DNA which will be transcribed and spliced into autonomous replicons in plant cells [25]. This not only effectively eliminates the need for generating RNA vectors by an* in vitro* process but also allows for all deconstructed viral vectors to be delivered by agroinfiltration. Since agroinfiltration can deliver vectors to most of the cells on the entire plant [15], the viral systemic spreading function is no longer needed. This alleviates the concern for transgene loss during systemic spreading and allows the deletion of coat protein to accommodate larger transgene insertion. Agroinfiltration also broadens the range of plant species susceptible to viral vector delivery beyond the natural virus hosts and allows for the delivery of vectors that are not mechanically transmissible in nature. Therefore, the development and application of deconstructed viral vectors not only overcome the limitations of full viral vectors but further enhance their versatility and transgene expression in transient systems.

The most commonly used deconstructed vectors rely on the MagnICON system, derived from TMV [26]. This system can be used either in the modular or the fully assembled form, depending on the application. The modular system facilitates simple cloning of the transgene because it is located in a separated plasmid of reasonable size. Furthermore, the transgene in the 3′ module can be paired with a suite of 5′ modules that contains different promoters and/or targeting sequences to various organelles. As a result, the modular MagnICON system provides the flexibility to test the expression of a transgene with different promoters and in different organelles by simply mixing different* A. tumefaciens* strains that carry various modules. Thus, it is best suited for the optimization of transgene expression and small scale RP production. In contrast, the fully assembled system sacrifices the flexibility to gain robustness for industrial scale production. It requires only one vector and one* A. tumefaciens* culture for agroinfiltration, greatly simplifying the upstream processing and reducing the overall cost of good. The MagnICON system has been tested at various production scales, from a few plants in a laboratory to 1.2 tons of plant material/day in industry. Collectively, they demonstrated that very high level accumulation of RPs (up to 5 mg per g leaf fresh weight (LFW)) can be achieved within 7–10 days after infiltration (dpi). They include RPs of all sizes and complexity, ranging from small subunit vaccines to large tetravalent antibodies [16, 17, 26–29].

**Figure 3:**
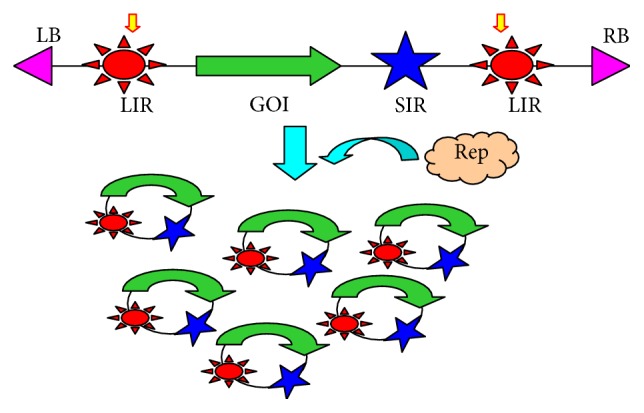
Geminiviral BeYDV vector for expression of recombinant proteins. The left (LB, pink triangle) and right border (RB, pink triangle) delineate the T-DNA construct that will be transferred into plant cells by* Agrobacterium*. Upon delivery into plant cells, expression of Rep gene produces the Rep protein (brown cloud) that nicks the LIRs (red stars) in the T-DNA to release a single-stranded DNA molecule (between the two yellow arrows). This DNA molecule recircularizes and is copied to make double-stranded DNAs that can replicate by the rolling circle mechanism to produce very high copy numbers of DNA templates (circles) and, in turn, abundant mRNAs of gene of interest (GOI) for the translation of the recombinant protein. Blue star: SIR; pink triangle: LB and RB of the T-DNA; red stars: LIRs; green arrow: gene of interest; brown cloud: Rep protein.

In spite of the success, the current MagnICON system cannot produce RPs with more than two heterosubunits. This problem is associated with the phenomenon called “competing replicons,” as codelivery of viral vectors built on the same viral backbone often results in early segregation and subsequent preferential amplification of only one of the vectors in a single cell [25]. For example, TMV and PVX are both competing viruses, but not with each other. If the heavy (HC) and light chain (LC) genes of a MAb are both cloned into the TMV or PVX vector, only one of the chains will be produced in a single cell and assembled MAb will not be produced. However, if the HC and LC gene are built on the TMV and PVX backbone, respectively, both chains can be expressed in the same cell, permitting their proper assembly into a functional MAb. [26]. This allows the MagnICON system to produce MAbs. However, identifying additional viruses that are noncompeting with both TMV and PVX for expressing proteins with three or more distinct subunits is a very difficult if not impossible task [20, 30]. We have circumvented this problem by developing a noncompeting vector system based on bean yellow dwarf virus (BeYDV), a monopartite virus in the Geminiviridae family [31]. Upon infection of plant cells, very high copy numbers of BeYDV genome are produced by rolling circle replication, which requires only one single viral protein (replication associated proteins (Rep)) (Figure 3) [31]. In the first generation of geminiviral vectors, the transgene and the Rep protein are supplied in two separate modules [19, 31]. Coagroinfiltration of the two modules resulted in high-level accumulation of RPs, which can be further increased by including a third module carrying a suppressor of gene silencing from TBSV (P19) [19]. For example, we showed that inclusion of the third P19 module increased the accumulation of hepatitis B core antigen (HBcAg) in* N. benthamiana* >4-fold [21]. Southern and Northern blot analyses indicated that codelivery of P19 only marginally increased the replicon copy number but greatly enhanced the accumulation of HBcAg-specific mRNA [21]. These results indicate that P19 indeed can increase target mRNA and protein accumulation, most likely by suppressing posttranscriptional silencing of the transgene. We then integrated the transgene, Rep, and P19 modules into a single vector system and demonstrated that the geminiviral system is noncompeting and permits the efficient expression and assembly of MAbs [32]. For large-scale manufacturing, we developed a single vector system that contains multiple replicon cassettes with each encoding for a distinct protein. Upon agroinfiltration into plant cells, each cassette was shown to assemble into an independent replicon and produces high levels of the protein/subunit it codes for, without competing with the replication of other replicons or the production of other proteins [32]. This single vector system obviates the need to generate several vector modules and manufacture multiple inocula of* A. tumefaciens* strains, further reducing capital and operational cost. Recently, a different geminiviral vector based on the mild strain of BeYDV-m has been developed and has shown its robustness in expressing two vaccine candidates [33]. These geminiviral systems may have broader plant host ranges than the MagnICON system [18, 19, 32, 34]. Overall, the geminiviral replicon system overcomes the difficulty of producing multiple heterosubunit proteins.

Other examples of deconstructed viral vectors include systems based on 5′ and 3′ untranslated regions (UTRs) of cowpea mosaic virus (CPMV) RNA-2 and tobacco yellow dwarf* Mastrevirus* (TYDV) [35, 36]. One version of the CPMV-based vectors is replication independent and, therefore, has great promise for agroinfiltration in plant hosts that are not compatible with replication-dependent vector systems. Excitingly, the CPMV-based vector has allowed the expression and assembly of Bluetongue VLPs in* N. benthamiana* that requires coexpression of four different protein components [30]. These plant-produced VLPs were shown to be immunogenic and provide protective immunity in sheep against a challenge of a Bluetongue virus field isolate, demonstrating the utility of this vector in producing complex and heteromultimeric proteins [30]. The TYDV-based vector system represents “bridge” vectors that allow the stable inheritance of the transgene and a robust yet controlled transient expression of a RP upon the induction with a specific chemical signal [36]. Plants are allowed to accumulate biomass in the growth phase while the integrated transgene remains silent and replicon amplification will be triggered upon induction for RP production. This type of bridge vector system effectively combines the strengths of both the stable and transient expression systems and potentially offers a complete platform for the rapid assessment of RP candidates and their transition to a large-scale commercial production.

### 3.3. Plant Hosts for Agroinfiltration

A prerequisite for a plant species to be successfully agroinfiltrated is its susceptibility to* A. tumefaciens* infection. Among susceptible plants, however, the amenability of different species to agroinfiltration varies significantly due to leaf structural differences in the cuticle, the density of stomata on the epidermis, and the compactness of mesophyll cells. Due to technological improvements, a rapidly expanding spectrum of plant species is now amenable for transgene delivery by agroinfiltration. Since transient expression systems for RP production do not generate transgenic plants, it does not have the risk of contaminating food crops or unintended transgene escape. This further expands plant hosts infected via agroinfiltration as an acceptable technology to the public and regulatory agencies. The choice of a particular plant host for protein expression is determined by its compatibility with available expression vectors, the nature of the target RP, and the scale of production.

#### 3.3.1. *Nicotiana* Hosts

The most popular host plant for agroinfiltration is* N. benthamiana* and related* Nicotiana* plants including tobacco. Besides being most amenable to agroinfiltration, these plants can produce large amount of biomass rapidly and are prolific seed producers for the industrial scale-up of production [20]. In addition, they are permissive to the replication of a variety of replicon-based vectors. The FDA and other regulatory agencies are familiar with clinical trial materials from these plant hosts, thus facilitating their acceptance in regulation-compliant processes [29, 37]. As a result, numerous RPs of various natures, sizes, and applications have been produced in* Nicotiana* hosts (Table 1). The examples below demonstrate the advantages and versatility of utilizing these plant hosts for agroinfiltration.

In a clinical trial,* Nicotiana* host-produced biologics were used to treat Non-Hodgkin's lymphoma (NHL). NHL is a group of blood cancers that is estimated to result in over 70,800 new cases in the USA alone in 2014. In NHL, each malignant B cell clone expresses a unique cell surface immunoglobulin (Ig) as the tumor-specific marker, making standard treatments ineffective. The variable nature of NHL calls for patient-specific cancer treatments that require an expression system with the flexibility to rapidly produce patient-specific vaccines. One of these vaccines consists of MAbs derived from each patient's own tumor. The cell culture-based production platforms do not have the speed and flexibility to produce these personalized vaccines. In contrast, plant production systems based on agroinfiltration can provide the optimal platform to meet this demand. Results showed that 20 patient-specific MAbs were produced at high levels in* N. benthamiana* leaves within two weeks of agroinfiltration [29]. The manufacturing process is robust, requiring only two weeks for MAb-based vaccine expression and purification and less than 12 weeks from biopsy to vaccination [29]. To test the safety and immunogenicity of the plant-expressed vaccine candidates, a phase I human clinical trial was initiated with 12 patients. Results indicated that the vaccine was well-tolerated without major side effects and 73% of the patients developed a tumor-specific immune response [37, 40]. This study demonstrated the rapidness and versatility of the agroinfiltration-based transient system in generating multiple patient-specific cancer vaccines and showcased the capacity of* N. benthamiana* in producing vaccines that are safe to administer and effective in the treatment NHL patients.

*Nicotiana* plant hosts were also utilized for commercial scale enzyme production. In the production of ethanol as a fuel extender, large quantities of cellulase are needed to saccharify cellulosic feedstocks. For more than 30 years, the high cost of cellulase from fungal fermentation has been a major impediment to the economic viability of cellulosic ethanol programs [48]. To reduce the cost of cellulase,* N. benthamiana* was used as a host to produce four cellulases for cell wall degradation via agroinfiltration [42]. Results showed that all four cellulases were expressed at high levels, up to 75% TSP [42]. Further analysis indicated that plant*-*produced cellulases are functional, efficiently converting cellulose to glucose [42]. Similar results were obtained between using syringe and spray agroinfiltration, indicating the scalability of the upstream process [42]. Importantly, the necessity of purification and costs associated therewith are avoided in the downstream processing, as the cellulases can be simply preserved at room temperature for up to four months in dehydrated* N. benthamiana* biomass as silage [42]. Technoeconomic analysis of a similar cellulase production system based on transgenic* N. tabacum* concludes that the plant-based system may offer a >30% reduction in unit production costs and an 85% reduction in the required capital investment compared with the current fungal-based fermentation system [49]. We speculate that the system based on spray agroinfiltration may result in a similar cost-saving benefit, presenting a system of cellulase production with unprecedented efficiency and cost-effectiveness. This process can find broad applications for production of other cost-sensitive RPs.

*N. benthamiana* hosts also offer opportunities to produce RPs with enhanced functionalities (biobetters). For example,* N. benthamiana* with “humanized” glycosylation pathways have been developed to enhance the safety and efficacy of plant-produced MAbs [50]. The difference in N-glycosylation between plant and mammalian-produced MAbs may alter the stability and/or efficacy of plant-produced MAbs or cause potential adverse effects through immune complex formation. To overcome this challenge, a double knockdown (ΔXF)* N. benthamiana* line was created to suppress the production of the two plant-specific glycans: *β*-1,2-xylose and core *α*-1,3-fucose [51]. Results indicated that anti-Ebola MAbs produced in the ΔXF plant line had no plant-specific N-glycans but contained the highly homogenous (90%) mammalian glycoform GnGn [52]. The lack of fucose and the high homogeneity of plant-derived MAbs have led to their higher affinity to the Fc receptor (FcgRIII) and their enhanced potency against Ebola virus over the mammalian cell-produced MAbs [52]. The superior potency of plant-produced MAbs was further demonstrated in a challenge study with nonhuman primates, in which plant-produced MAbs were far more protective against a lethal Ebola challenge than those produced in mammalian cells [45]. In a remarkable and exciting development, these plant-made MAbs were recently used to treat two American Ebola patients and showed promising results [53]. Similarly, our studies showed that ΔXF plant-derived anti-WNV MAbs displayed enhanced viral neutralization in comparison to their mammalian counterparts [28]. These new* N. benthamiana* hosts are being applied to produce biobetter RPs beyond the realm of MAbs.

#### 3.3.2. Non-*Nicotiana* Hosts

Certain RPs require a non-*Nicotiana* plant host for their optimal expression. Fortunately, improvements in technologies have allowed the application of agroinfiltration to many plant species beyond* Nicotiana *plants, including lettuce, tomato, alfalfa, petunia, potato, cotton, grapevine, switchgrass, radish, pea, lupine, flax, citrus, lentil, sunflower, and* Arabidopsis* [11]. Agroinfiltration methods have also been applied to woody trees including aspen, poplar, birch, eucalyptus, pines, and spruces [13]. In addition to leaf tissue, petals of tobacco, petunia,* Antirrhinum majus*,* Gerbera jamesonii*, several species of* Dendrobium* flowers, and the fruits of tomatoes and strawberries have also been successfully agroinfiltrated with transgene constructs [54].

Among these options, lettuce is a prime example to demonstrate the special utility of non-*Nicotiana* hosts in producing RPs. Despite the aforementioned advantages of* Nicotiana* hosts, they do produce unusually higher levels of phenolics and alkaloids than other plant species. These compounds foul purification resins and are difficult to remove from the target RP in downstream processing, adding to production resources and costs [55, 56]. This is especially problematic for RPs with pharmaceutical applications, as they need to be free of these plant compounds to meet regulations of the FDA. Thus, there is a need to identify plant hosts that produce lower levels of phenolics and alkaloids yet retain the robustness of RP production. Lettuce (*Lactuca sativa*) is already cultivated commercially in large scales and its yield and speed of biomass generation can easily match those of* Nicotiana* plants. Lettuce produces negligible quantities of phenolics and alkaloids and thus would overcome the challenge of their removal during downstream processing. Agroinfiltration with nonviral vectors indicated that lettuce expresses a variety of functional RPs, albeit the expression levels were low [57, 58]. To further demonstrate the potential of lettuce as a host for RP production, we explored the use of deconstructed viral vectors to express pharmaceutical proteins in lettuce (Table 1). We first examined the expression of a VLP vaccine candidate for norovirus based on the capsid protein of Norwalk virus (NVCP) with geminiviral vectors. NVCP was expressed at levels which are comparable with that in* N. benthamiana*, at the highest expression levels ever reported for a vaccine in lettuce [34]. Furthermore, lettuce-produced NVCP efficiently assembled into VLPs with a diameter typical of native NVCP VLPs [21, 34]. Moreover, these VLPs are fully functional and can induce potent immune response in mice [21]. These studies demonstrated that lettuce is as robust as* Nicotiana* plants for RP production with agroinfiltration. Beyond that, this study also demonstrated the superiority of lettuce over* Nicotiana* plants for expressing VLPs. Owing to their structural resemblance to native viruses but lacking infectious viral genomes, VLPs have been shown to have tremendous potential in immunogenicity, multivalency and safety as vaccines against many diseases [20]. However, the porous and dynamic nature of the VLP structure also makes it susceptible to trap contaminant molecules inside. As a result, it is a very difficult task to remove plant secondary metabolites from the feedstream of* Nicotiana* plant-produced VLPs [16]. If not resolved, this problem will diminish the vast potential of VLPs and plant transient expression technology. Due to the low level of secondary metabolites in lettuce tissue, NVCP VLPs can be purified to high purity from lettuce extracts without the extra need for elimination of phenolics and alkaloids [34].

The advantage of lettuce has also been showcased for the production of MAbs, another group of proteins with high pharmaceutical relevance. MAbs can be efficiently produced in several plant hosts. For example, MAbs against Ebola virus and WNV can be expressed at very high levels in* N. benthamiana* with MagnICON and geminiviral vectors [17, 32]. These plant-produced MAbs are fully functional in protecting animals from lethal challenge of viral infections [17]. In addition to the native MAbs, large MAb variants such as tetravalent antibodies and recombinant immune complexes have been successfully produced in* N. benthamiana* [27, 28, 41, 43]. The most popular and efficient method of purifying MAbs is Protein A affinity chromatography. As a RP itself, Protein A is costly and can be easily damaged by a cleaning reagent. Unfortunately, phenolics and alkaloids in* N. benthamiana* feedstream foul Protein A resins and are hard to remove from the target MAb. Consequently, extra purification steps are required to remove these secondary metabolites from the feedstream before loading to Protein A resin [17]. Moreover, frequent cleaning of resins with harsh reagents is necessary to prevent fouling [59]. These extra measures complicate downstream processing, shorten the life span of protein A, and add extra capital and operational cost for MAb production. Our results indicate that the ammonium sulfate precipitation step for MAb purification, which is partially responsible for removing secondary metabolites from tobacco extract, can be bypassed due to the negligible amounts of plant compounds in the lettuce feedstream [18, 34]. Thus, lettuce extracts containing MAbs can be directly loaded onto the Protein A column, avoiding resin fouling concerns [18, 34]. As a result, the life cycle of Protein A resin is prolonged and the overall production cost of MAbs is reduced.

Our success in producing functional VLPs and MAbs with commercially produced lettuce demonstrated another advantage of lettuce as a host for large-scale agroinfiltration. Since agricultural and food industries have already established the infrastructure and technology needed for large-scale lettuce growing and processing, they can be rapidly adapted for the production of RPs. This suggests that biomass production could be subcontracted to existing commercial growers. This will forego the need for capital investment of purpose-built biomass facilities but allows access to potentially unlimited quantities of inexpensive plant material for large-scale manufacturing of RPs. Besides lettuce, other plant species are being explored as hosts for agroinfiltration to allow for the production of RPs with unique properties.

## 4. Conclusions

The development of deconstructed viral vectors has reinvigorated the field of plant-made RPs and provided a production platform with superior protein yield, speed, scalability, versatility, safety, and cost-saving benefits. A recent breakthrough in plant glycoengineering allows plants to produce RPs with tailor-made N-glycans and expands the utility of plants in developing biobetters with superior functional and safety profiles. The lack of a scalable technology to deliver transgene into plant cells was one of the remaining hurdles for the commercial application of plant transient systems. As discussed in this review, this challenge has been overcome effectively by various agroinfiltration technologies. We believe that further optimization of agroinfiltration technologies will expedite the acceptance of plant transient expression systems for the commercial production of a broad range of RPs.

## Figures and Tables

**Table 1:** Examples of recombinant proteins produced in plants by agroinfiltration.

Plant host	Vector	Biologic target	Development stage	References
*N. benthamiana *	Nonviral vector	Influenza A H5N1 HA VLP vaccine	Phase I/II human trial	[38, 39]
*N. benthamiana *	TMV	NHL personalized vaccine	Phase I human trial	[37, 40]
*N. benthamiana *	CPMV	BTV 4-component VLP vaccine	Preclinical	[30]
*N. benthamiana *(WT, ΔXF)	TMV/PVX	Tetravalent antibody WNV therapeutic	Preclinical	[28, 41]
*N. benthamiana *	TMV	Cellulases for ethanol production	Early development	[1, 42]
*N. benthamiana *	TMV/PVX	Ebola immune complex-based vaccine	Preclinical	[27, 43]
*N. benthamiana *	TMV, BeYDV, CPMV	HBcAg nonenveloped VLP vaccine	Preclinical	[19, 44]
*Lettuce, N. benthamiana *	TMV/PVX, BeYDV	Ebola therapeutics based on MAb	Preclinical	[18, 32, 34, 45]
*Lettuce, N. benthamiana *	TMV, BeYDV	Norovirus NVCP VLP vaccine	Preclinical	[19, 34, 46]
*Lettuce, N. benthamiana *	TMV/PVX, BeYDV	WNV therapeutics based on MAb	Preclinical	[17, 28, 34]
*Lettuce, N. benthamiana *(WT, ΔXF)	TMV, BeYDV	WNV DIII vaccine	Preclinical	[31, 34, 47]

HA: hemagglutinin; VLP: virus-like particle; NHL: non-Hodgkin's lymphoma; BTV: bluetongue virus; WT: wild-type; ΔXF: plants with double knockdown of *β*-1,2-xylose and core *α*-1,3-fucose; WNV: West Nile virus; HBcAg: hepatitis B core antigen; MAb: monoclonal antibody; NVCP: Norwalk virus capsid protein; DIII: domain III of envelope protein.

## Abbreviations

RP: Recombinant protein
GMO: Genetically modified organism
cGMP: current good manufacturing practice
TSP: Total soluble protein
LFW: Leaf fresh weight
dpi: Days postinfiltration
BeYDV: Bean yellow dwarf virus
TMV: Tobacco mosaic virus
PVX: Potato virus X
TBSV: Tomato busy stunt virus
VLP: Virus-like particle
NHL: Non-Hodgkin's lymphoma
WNV: West Nile virus
BTV: Bluetongue virus
MAb: Monoclonal antibody.
